# Haploinsufficiency of EHMT1 improves pattern separation and increases hippocampal cell proliferation

**DOI:** 10.1038/srep40284

**Published:** 2017-01-10

**Authors:** Marco Benevento, Charlotte A. Oomen, Alexa E. Horner, Houshang Amiri, Tessa Jacobs, Charlotte Pauwels, Monica Frega, Tjitske Kleefstra, Maksym V. Kopanitsa, Seth G. N. Grant, Timothy J. Bussey, Lisa M. Saksida, Catharina E.E.M. Van der Zee, Hans van Bokhoven, Jeffrey C. Glennon, Nael Nadif Kasri

**Affiliations:** 1 Department of Cognitive Neuroscience, Radboudumc, Donders Institute for Brain, Cognition and Behaviour, Nijmegen, the Netherlands; 2 Synome Ltd., Babraham Research Campus, Cambridge CB22 3AT, UK; 3 Department of Psychology, University of Cambridge, Cambridge, UK; 4 Behavioural and Clinical Neuroscience Institute, University of Cambridge, Cambridge, UK; 5 Neuroscience Research Center, Institute of Neuropharmacology, Kerman University of Medical Sciences, Kerman, Iran; 6 Department of Human Genetics, Radboudumc, Donders Institute for Brain, Cognition, and Behaviour, Nijmegen, The Netherlands; 7 Centre for Clinical Brain Sciences, Chancellors Building, University of Edinburgh, 49 Little France Crescent, Edinburgh EH16 4SB, UK; 8 Department of Cell Biology, Radboudumc, Radboud Institute for Molecular Life Sciences, Nijmegen, The Netherlands

## Abstract

Heterozygous mutations or deletions of the human Euchromatin Histone Methyltransferase 1 (*EHMT1*) gene are the main causes of Kleefstra syndrome, a neurodevelopmental disorder that is characterized by impaired memory, autistic features and mostly severe intellectual disability. Previously, *Ehmt1*^+/−^ heterozygous knockout mice were found to exhibit cranial abnormalities and decreased sociability, phenotypes similar to those observed in Kleefstra syndrome patients. In addition, *Ehmt1*^+/−^ knockout mice were impaired at fear extinction and novel- and spatial object recognition. In this study, *Ehmt1*^+/−^ and wild-type mice were tested on several cognitive tests in a touchscreen-equipped operant chamber to further investigate the nature of learning and memory changes. Performance of *Ehmt1*^+/−^ mice in the Visual Discrimination & Reversal learning, object-location Paired-Associates learning- and Extinction learning tasks was found to be unimpaired. Remarkably, *Ehmt1*^+/−^ mice showed enhanced performance on the Location Discrimination test of pattern separation. In line with improved Location Discrimination ability, an increase in BrdU-labelled cells in the subgranular zone of the dentate gyrus was observed. In conclusion, reduced levels of EHMT1 protein in *Ehmt1*^+/−^ mice does not result in general learning deficits in a touchscreen-based battery, but leads to increased adult cell proliferation in the hippocampus and enhanced pattern separation ability.

Haploinsufficiency of the Euchromatic histone methyltransferase 1 (*GLP/EHMT1*) gene is the cause of Kleefstra syndrome (KS; #OMIM 610253)^1^. The core features of KS are intellectual disability (ID), hypotonia and cranio-facial abnormalities, frequently associated by various other conditions as congenital (heart) defects, epilepsy and Autism Spectrum Disorder (ASD)^1^,^2^. The effects of mutations in the *Ehmt* gene studied in *Drosophila* (EHMT) and in mouse models (*Ehmt1*) at molecular, cellular and behavioural levels validated some core features of the Kleefstra syndrome^3^,^4^,^5^,^6^,^7^,^8^,^9^,^10^,^11^.

We previously reported that haploinsufficiency of the *Ehmt1* gene can result in altered performance on several classical tests of learning and memory^4^. *Ehmt1*^+/−^ mice show reduced novelty preference in both novel object and spatial recognition tests, indicating putative perirhinal cortex- and hippocampus-dependent memory impairments. However, *Ehmt1*^+/−^ mice showed normal performance on a spatial navigation task (Barnes maze) and exhibited increased freezing during acquisition of a cued fear-conditioning paradigm and during the test phase of contextual fear conditioning. Finally, *Ehmt1*^+/−^ mice were impaired at cued fear extinction. These findings may be, in part, explained by putative differences in anxiety- and exploration-related behaviour^5^,^11^. Therefore, a thorough characterization of the learning and memory ability of *Ehmt1*^+/−^ mice in low-aversive and low-stress tests, using positive reinforcers, would improve our insight into the cognitive phenotype of this mouse model of intellectual disability.

Here we tested *Ehmt1*^+/−^ and litter-matched wild-type (WT) mice on several learning and memory tests in a touchscreen-equipped operant chamber^12^. This cognitive testing method requires animals to respond to visual stimuli by touching a touch-sensitive screen in order to receive an appetitive reward. It imposes a low demand on the motor ability or innate novelty driven exploration of animals and is relatively non-aversive. Due to the flexible nature of this method, animals can be tested on several cognitive domains within the same setup, allowing for between-task comparison. In this study, six different tests of learning and memory were implemented (Fig. 1), some of which also assessed executive functions such as response inhibition and behavioural flexibility^13^,^14^,^15^. In addition, animals were tested on the Location Discrimination task^13^,^16^ in order to assess spatial learning and, in particular, pattern separation^17^.

Although we could confirm lower levels of spontaneous activity in the *Ehmt1*^+/−^ mice compared to WT mice, learning and memory performance of *Ehmt1*^+/−^ mice was mostly unimpaired. Interestingly however, *Ehmt1*^+/−^ mice performed better on the location discrimination task, in particular when similarity of locations was high. Using the location discrimination task, it has been shown that adult born neurons in the hippocampal dentate gyrus (DG) are involved in pattern separation^18^. Furthermore, this touchscreen task was found to be sensitive to changes in adult hippocampal neurogenesis in different animal models, both in the case of increased adult neurogenesis^19^ and decreased adult neurogenesis^20^. Accordingly, in *Ehmt1*^+/−^ mice, we found increased neural stem cell proliferation in the subgranular zone of the DG as assessed by BrdU incorporation.

## Results

### *Ehmt1* ^+/−^ mice show reduced activity but normal learning and memory in the touchscreen operant chamber

**Figure 1:**
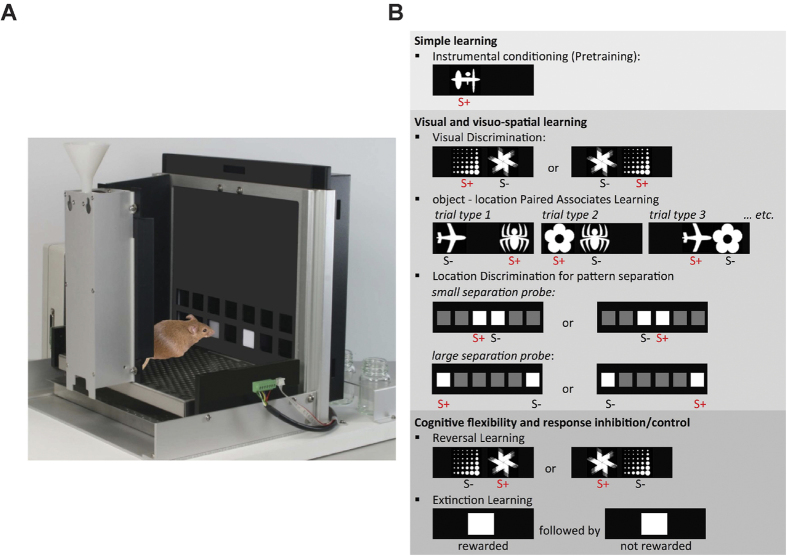
Cognitive testing in the touchscreen operant chamber. (**A**) Example of a Campden touchscreen operant chamber for mice. (**B**) Overview of the cognitive tasks: *Ehmt1*^+/−^ and WT mice were tested on operant conditioning (Pretraining); Visual Discrimination; object-location Paired-Associates Learning; Reversal Learning; Extinction Learning and Location Discrimination for pattern separation. (Panel B; Adapted by permission from Macmillan Publishers Ltd: Nature Neuroscience, copyright (2013)^46^ and Cold Spring Harbor Laboratory Press, copyright (2013)^12^).

To test cognitive function of *Ehmt1*^+/−^ mice in low-aversive and low-stress conditions with the use of positive reinforcers, we tested *Ehmt1*^+/−^ and WT mice on several learning and memory tests in touchscreen-equipped operant chambers (for an overview, see Fig. 1 and Figs S1–4).

**Figure 2:**
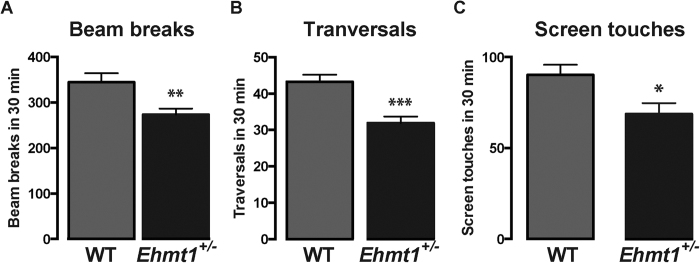
Measures of activity during the first exposure of animals to the touchscreen chamber. (**A**) *Ehmt1*^+/−^ mice (n = 23) are hypoactive as they make less (**A**) Beam breaks (p = 0.007) (**B**) Chamber traversals (p < 0.001) and (**C**) Screen touches (p = 0.011) when compared to WT mice (n = 25).

Cognitive testing was preceded by habituation to the testing chambers, during which activity registration was performed. In accordance with earlier findings^5^,^11^, *Ehmt1*^+/−^ mice exhibited hypoactivity in the novel environment reflected, in this instance, by a significantly lower number of beam breaks (U = 149.5, p = 0.007), chamber traversals (t_46_ = 4.383, p < 0.001) and screen touches (t_46_ = 2.635, p = 0.011) compared to their WT counterparts on the first day of habituation (Fig. 2A–C). This was replicated in the second cohort of animals tested for behaviour in which *Ehmt1*^+/−^ mice made significantly fewer chamber traversals compared to WT mice (WT 36.79 ± 2.17; *Ehmt1*^+/−^ 29.39 ± 1.10; p = 0.034).

Next, we tested learning and memory abilities of *Ehmt1*^+/−^ mice. Touchscreen testing commenced with instrumental acquisition (pretraining). This was followed by the visual discrimination & reversal-learning task, which was implemented to assess more complex learning and cognitive flexibility. Thereafter, mice were subjected to object-location paired-associates learning task to study their visuospatial learning ability and after this, animals were trained on a simple instrumental response followed by the extinction learning task to study response inhibition. Firstly, *Ehmt1*^+/−^ mice did not differ from WT animals with respect to the average reward collection latency (p > 0.1) and response latencies (p > 0.1). As for cognitive performance, *Ehmt1*^+/−^ mice required a similar amount of trials to reach criterion on pretraining, visual discrimination & reversal learning, object-location paired-associates learning and extinction learning tasks (see Table 1, Figs S1–4). During the early phase of reversal learning, *Ehmt1*^+/−^ mice were found to have a higher perseverative index, which may be indicative of reduced cognitive flexibility, however this did not affect the rate of reversal acquisition. Overall, we confirmed that *Ehmt1*^+/−^ mice showed reduced spontaneous activity in a novel environment. Furthermore, *Ehmt1*^+/−^ mice did not exhibit gross impairments in their general learning and memory abilities compared to their WT control counterparts.

### Enhanced location discrimination ability in *Ehmt1* ^+/−^ mice

**Figure 3:**
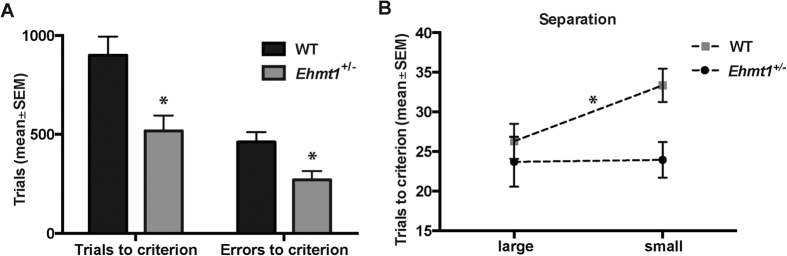
Location Discrimination performance. (**A**) The number of trials and errors animals required to reach the performance criterion during the initial acquisition phase at an intermediate distance. (**B**) Performance on Location Discrimination large separation and small separation probe sessions. *Ehmt1*^+/−^ (n = 8) mice outperform WT mice (n = 17), as they needed on average fewer trials to reach the within-session criterion of 7 correct out of 8 consecutive responses (main effect F(1,25) = 6.36; p = 0.019).

In addition to the learning and memory tests described above, a separate cohort of *Ehmt1*^+/−^ mice and WT animals was subjected to the location discrimination task of pattern separation. In this task, animals were required to discriminate and choose between two square response windows. Distance between the locations was varied (i.e. small, intermediate, large), which resulted in increased or decreased similarity of these locations. The rationale behind this is that increased similarity of the to-be-remembered stimuli places a higher demand on resolving confusability between input representations and thus requires, at the network level, increased pattern separation of such inputs^13^. Putative impairments of location discrimination at a small distance (but not at a large distance) would be interpreted as a pattern separation impairment. Surprisingly, *Ehmt1*^+/−^ mice outperformed their WT counterparts during location discrimination acquisition at intermediate distance. The *Ehmt1*^+/−^ group required significantly fewer trials and made fewer errors to reach the overall acquisition criterion (Fig. 3A). Pattern separation probe sessions (using small or large distance between locations) revealed that *Ehmt1*^+/−^ mice required fewer trials to reach the within-session acquisition criterion on such sessions. This seemed to be particularly pronounced on sessions in which animals were required to choose between stimuli at a small separation distance (Fig. 3B). A main effect of genotype on probe session performance was found F(1,25) = 6.36; p = 0.019), but no genotype × separation interaction F(1,25) = 1.19; p = 0.286). No differences were found in the average reward collection latency (*Ehmt1*^+/−^ mice 1.7 ± 0.2 s; WT mice 1.7 ± 0.1 s) or response latencies (correct response latencies: *Ehmt1*^+/−^ mice 7.4 ± 1.3 s; WT mice 7.6 ± 0.7 s; incorrect response latencies: *Ehmt1*^+/−^ mice 6.7 ± 0.9 s; WT mice 6.9 ± 0.6 s, all p > 0.1). During the recall-test, *Ehmt1*^+/−^ mice performed similar to WT mice when assessed for their memory of the rewarded location across a 72-h delay (Fig. S5). To summarize, *Ehmt1*^+/−^ mice showed enhanced acquisition of the location discrimination task and enhanced performance on post-acquisition pattern separation probe sessions. The improvement was most pronounced on sessions in which stimuli were presented at a small or intermediate distance, indicating enhanced pattern separation ability.

### Increased neural stem cell proliferation in the subgranular zone of the dentate gyrus of *Ehmt1* ^+/−^ mice

**Figure 4:**
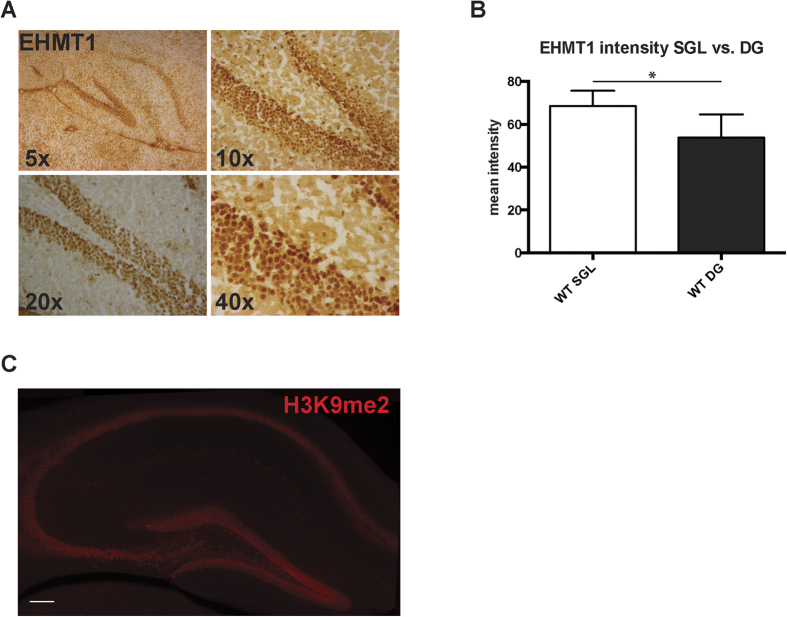
EHMT1 expression pattern in the dentate gyrus. (**A**) EHMT1 staining intensity in the DG at different levels of magnification (**B**) Quantification of EHMT1 staining intensity in the SGZ (n = 30 cells) and DG (n = 30 cells). (**C**) Representative H3K9me2 immunolabeling in a hippocampal section (Scale bar: 100 μm).

**Figure 5:**
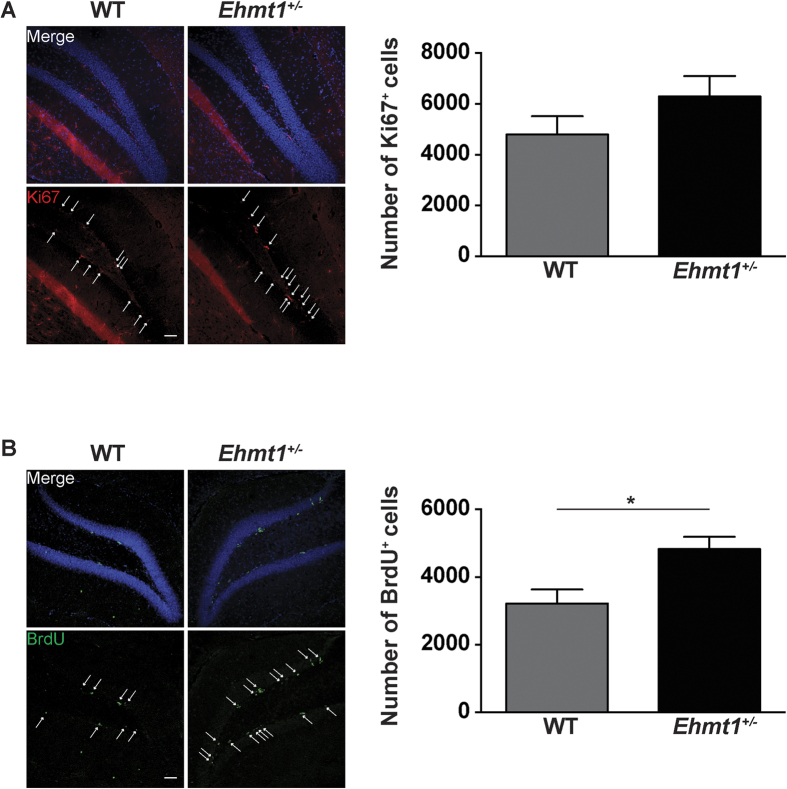
Increased cell proliferation in the dentate gyrus of *Ehmt1*^+/−^ mice. (**A**) Ki67 immunoreactivity (red) in the DG SGZ. White arrows indicate the Ki67^+^ cells. (**B**) Total quantification of Ki67^+^ cells (WT = 8; *Ehmt1*^+/−^ = 7), p = 0.187 (**C**) BrdU immunolabeling (green) in the DG SGZ. White arrows indicate the BrdU^+^ cells. (**D**) Total amount of BrdU^+^ cells (WT n = 8 animals; *Ehmt1*^+/−^ n = 7 animals), p = 0.012. For all images scale bar: 20 μm.

Performance in the location discrimination task, particularly on trials which pose a high demand on pattern separation ability, was previously found to depend on adult neurogenesis in the hippocampal dentate gyrus (DG)^18^. Additionally, increased location discrimination performance has been correlated with enhanced adult neurogenesis^19^. Given the better performance of *Ehmt1*^+/−^ mice in this task compared to WT controls, we assessed whether *Ehmt1* haploinsufficiency led to an alteration in the number of proliferating neural stem cells in the DG SGZ. To do this, we first analyzed the expression pattern of EHMT1 in the DG subgranular zone (SGZ) (Fig. 4A). We observed that EHMT1 immunostaining intensity levels were significantly higher in the SGZ cells compared to the cells in the granular cell layer of the DG (Fig. 4B) (SGZ = 68.55 ± 3.20 a.u.; DG = 53.85 ± 4.83 a.u.; n = 5–5; *t* = 2.53, p = 0.035, T-test). Interestingly, H3K9me2 expression pattern paralleled the intensity levels of EHMT1, which together with G9a/EHMT2 are responsible for the catalysis of H3K9me2 (Fig. 4C)^21^,^22^. The increase in EHMT1 levels in the SGZ prompted us to test whether EHMT1 is involved in the regulation of cell proliferation in the DG. To this end, we quantified the number of cells in the SGZ that were positive for Ki-67 (Ki-67^+^), a marker of proliferating cells. Although we observed a nominal increase in the amount of Ki67^+^ cells in *Ehmt1*^+/−^ mice, this was not significant (Fig. 5A), (WT mice = 4796 ± 721 cells; *Ehmt1*^+/−^ mice = 6296 ± 803 cells; n = 8 and 7 animals respectively; *t* = 1.392, p = 0.187, T-test). Ki-67 is a proliferative marker expressed during G1, S and G2 phase of the cell cycle. Although Ki-67 expression is informative, its temporal resolution is not precise enough to give information about the actual pool of cells with replicating DNA. To gain more insight we used BrdU labeling, since this marker only labels the cells that are actively replicating DNA^23^. WT and *Ehmt1*^+/−^ P70 mice were injected with BrdU (50 μg/g) four times every 4 h during a 12 h period and animals were sacrificed 24 h later. BrdU^+^ cells were quantified using serial sections as described previously^24^. *Ehmt1*^+/−^ mice had a higher number of BrdU^+^ cells compared to WT animals (Fig. 5B) (WT = 3216 ± 415 cells; *Ehmt1*^+/−^ = 4826 ± 361 cells; n = 8 and 7 animals respectively; *t* = 2.882, p = 0.012, T-test). Collectively, these experiments suggest that EHMT1 is involved in regulating proliferation of neural stem cells in the DG SGZ.

## Discussion

We show here that EHMT1 haploinsufficient mice perform normally on several tests of learning and memory in a touchscreen-equipped operant chamber. *Ehmt1*^+/−^ mice acquired visual discrimination & reversal learning, object-location paired-associates learning and extinction learning to the same level and at the same rate as did WT controls. *Ehmt1*^+/−^ mice did show signs of reduced flexibility, as reflected in an increased perseveration index during the early phase of reversal learning. However, this did not impact overall acquisition speed of the reversed contingencies and perseverative behaviour did not differ during location discrimination training, which also contains a reversal component. It can therefore be concluded that *Ehmt1*^+/−^ mice are generally unimpaired at learning and memory tasks when tested in a low-stress and unaversive environment, using highly translatable cognitive tests. This is quite remarkable, as patients with Kleefstra syndrome have in most cases severe intellectual disability^25^ and exhibit significant behavioral and psychiatric problems including anxiety (personal communication). It is possible that impairments found earlier in classical learning and memory tests^4^ such as novel object (and object in place) recognition memory and fear conditioning, could be partially driven by enhanced anxiety in *Ehmt1*^+/−^ mice^5^ and to a lesser extent by impaired cognitive ability. Extended training time during operant conditioning may therefore be beneficial for cognitive performance of *Ehmt1*^+/−^ mice as it ensures thorough habituation to the testing setup. It is however interesting to note that although most Kleefstra patients show severe intellectual disability, recent next generation sequencing studies in autism cases has identified a patient with loss of function mutations in EHMT1 with normal intellectual performance^26^. The *Ehmt1*^+/−^ mouse model, which shows clear autistic features might therefore represent a face valid model for the cases of KS that are associated with autism compared to intellectual disability. No genotype phenotype link has however been identified in patients thus far, but with the increasingly available sequencing techniques, it is expected that the phenotypic spectrum will widen and reveal individuals with milder cognitive phenotypes.

Comparing cognition in mice and humans has traditionally been difficult. The development of touchscreen test for rodents now makes it possible to perform the same test in both species. In a recent study, mice lacking the *Dlg2* gene (*Dlg2*^−/−^) and individuals with *DLG2* CNV deletions were tested in the same touchscreen test for object-location paired-associates task^27^. Interestingly, both mice and humans lacking the *DLG2* gene showed impaired performances. It will therefore be interesting to compare the observations in this study with the results from Cambridge Neuropsychological Test Automated Battery, (CANTAB) performed on Kleefstra syndrome patients.

As for the enhanced performance of *Ehmt1*^+/−^ mice compared to WT controls on the location discrimination task, this may indicate an improved DG-dependent pattern separation function^16^,^13^. Previously, pattern separation ability (as assessed by location discrimination performance), was shown to be sensitive to disruptions in adult hippocampal neurogenesis^18^,20,^28^, whereas enhanced performance was found to correlate with increased NSC proliferation in mice housed with a running wheel^19^. In line with this, we found that adult *Ehmt1*^+/−^ mice have higher levels of cell proliferation in the DG, as measured by the number of BrdU^+^ cells in the SGZ. Although this was not paralleled by a significant increase in the number of Ki67^+^ cells, there was a nominal trend for a higher number of cells in *Ehmt1*^+/−^ mice. While both Ki67 and BrdU are considered *bona fide* markers of cell proliferation^23^, Ki67 is expressed during most cell cycle phases, potentially giving a less precise estimation of the actual pool of cells that are replicating the DNA. This is in contrast with BrdU, which is only incorporated into DNA during the S-phase^29^. Enhanced proliferation levels in the *Ehmt1*^+/−^ mice are in line with the fact that EHMT1 likely restricts proliferation of neural stem cells in the SGZ and with its higher expression in the SGZ demonstrated in this study. Several lines of evidence suggest that EHMT1, together with its partner EHMT2, are required to restrict developmental genetic programs for correct cell proliferation and cell differentiation^30^,^31^,^32^. Interestingly, the lack of EHMT1/EHMT2 was shown to result in a failure of cells to undergo cell commitment and increased proliferation of human hematopoietic stem cells, brown adipose cells and retinal layering^33^,^34^,^35^,^36^. Moreover, EHMT1/2 interacts with PRC2 (Polycomb complex) to repress and prevent re-expression of time developmental genes^37^. In this regard, it is interesting to note that *Ezh2*^−/−^ mice, a known component of PRC2 complex, showed a higher number of BrdU^+^ cells in the developing cerebral cortex^38^. Brain-derived neurotrophic factor (BDNF) might be another potential mechanism to explain improved pattern separation performance. We recently showed that BDNF levels are abnormally increased in the *Ehmt1*^+/−^ mice^39^. This is interesting since BDNF positively modulates neural stem cell proliferation and recently has been shown to be necessary for pattern separation in rats^40^,^41^,^42^,^43^.

Further studies are however needed to clarify the role of EHMT1 in the generation of new neurons in the DG, and whether this histone methyltransferase is involved in regulating the balance between self-renewal and differentiation of neurons or glial cells. Furthermore, this would provide valuable information about the cellular mechanisms that underlie Kleefstra syndrome.

## Materials and Methods

### Animals

For the experiments presented in this study, mice heterozygous for a targeted loss-of-function mutation in the *Ehmt1* gene (*Ehmt1*^+/−^ mice) and their WT littermates on C57BL/6 background were used, as previously described^5^. Animal experiments were performed at two sites, the Radboudumc (Nijmegen, The Netherlands) and the University of Cambridge (Cambridge, UK). Each test was done entirely at one site or completely at both sites, when done in duplicate. Mice used in experiments executed at the Radboudumc (Nijmegen, The Netherlands) were bred in-house. For behavioural experiments performed at the University of Cambridge, male mice from Radboudumc (Nijmegen, The Netherlands) were rederived into the Biological Services Unit of the Babraham Research Campus (Cambridge, UK) on a C57BL/6^Babr^ background. Males bearing the mutation were then crossed with C57BL/6^Babr^ WT females to generate the mice used in the present study. These mice were transferred to the animal facility of the University of Cambridge (Cambridge, UK) for behavioural testing around 8 weeks of age. Only males were used for all the experimental procedures here described. Male mice were housed in standard size cages (396mm w × 215mm d × 172 mm h) containing a plastic shelter and enrichment material in a temperature and humidity-controlled room under a 12 h light/dark cycle (lights off at 7.00 am). All procedures involving animal experimentation and experimental protocols were carried out in accordance and were approved by the Animal Care Committee of the Radboudumc, the Netherlands, conforming to the guidelines of the Dutch Council for Animal Care and the European Communities Council Directive of 24 November 1986 (86/609/EEC) or were executed in accordance with the United Kingdom Animals (Scientific Procedures) Act (1986).

### Cognitive testing in the touchscreen operant chamber

#### Experimental design

*Ehmt1*^+/−^ mice and their WT littermates were tested using touchscreen-equipped operant chambers, which make use of positive reinforcement (liquid or food reward). Two cohorts of mice were used, which were tested during daily, 1 h sessions. As a rule, mice were tested 5 times per week, but this occasionally varied between 4–7 times per week. Sessions occurred during the first half of the active (dark) phase, for several months. The first cohort was tested at the University of Cambridge and was composed of 25 WT and 23 *Ehmt1*^+/−^ mice that were 12 weeks of age when testing began. Mice were first subjected to a simple measure of activity registration, which occurred during the first habituation session. Following this, 13 WT and 10 *Ehmt1*^+/−^ mice were given further testing. After pretraining, these mice were subjected to the following learning and memory tasks: Visual Discrimination and Reversal learning, object-location paired-associates learning and extinction learning, consecutively. A second cohort of mice was tested at the Radboudumc (Nijmegen, Netherlands). This cohort was composed of 19 WT and 10 *Ehmt1*^+/−^ mice that were 11 weeks old at the start of testing. Similar to the first cohort, mice were first tested on a simple measure of activity and subjected to pretraining. This was followed by the location discrimination task for spatial pattern separation, which consisted of an initial acquisition phase followed by pattern separation probe-sessions. In order to ensure that animals were motivated to perform the task for a food reward, all mice were subjected to mild food restriction to 85–90% of their free feeding body weight.

#### Apparatus

Automated touchscreen operant chambers were used of two similar types; Bussey-Saksida touchscreen chambers (Campden Instruments, Loughborough, UK) and testing chambers assembled from separate parts (Med Associates, Inc., St. Albans, VT, USA) in-house. For a detailed description of both types of boxes, see Horner *et al*.^14^. In short, both types of chambers consisted of a modular chamber made out of black Perspex walls and a metal grid floor within a sound-attenuating box. A reward tray, equipped with a tray-light, was situated opposite to the screen in which either strawberry milkshake (Campden) or sugar pellets (Med-Associates) was delivered. Chambers were equipped with a light on the ceiling (house-light, 3 W), a tone generator and a touch-sensitive screen using infrared photocells. Campden chambers were additionally equipped with infrared beams at the front and back of the chamber for activity registration. A black plastic mask with response windows was used to cover the touchscreen during testing, dimensions of which were dependent on the specific task.

#### Learning and memory tests

For an overview of the tests see Fig. 1 and a short description below. Detailed protocols used in this study are available elsewhere^13^,^14^,^15^. Pretraining was utilized to train mice to touch the screen for a reward and familiarize animals to common trial elements: a training session (maximum 1 h) consisted of 30 trials and to start each trial, mice were required to nose-poke in the reward tray to “initiate” stimulus presentation on the screen. Upon making a correct response, touching the screen resulted in the delivery of a food reward and the sound of a tone (secondary reinforcer). An incorrect response resulted in a time-out (5 s) and the presentation of a correction trial (i.e. the same stimulus and stimulus location) until a correct response was made. Correction trials were used to prevent the formation of a stimulus- or side-bias and were also utilized in the visual discrimination and reversal learning task and the paired-associates learning task. Trials were separated by an inter-trial interval lasting 20 s. The first two pretraining sessions consisted of habituation to the testing chamber, during which activity registration was performed by recording the number of infrared beam breaks at the front and back of the chamber. Pretraining preceding the visual discrimination task was performed using a 2-hole mask and a range of 40 visual stimuli (randomly presented one at a time, in one of the two locations), whereas pretraining for the location discrimination task was performed without a mask and using a white square presented in 1 of 6 locations.

For visual discrimination and reversal learning^14^,^15^, mice had to discriminate between two stimuli: “fan” (correct, S+) and “marbles” (incorrect, S-), (see Fig. 3b)^14^, presented in a pseudorandom location. Mice were considered to have acquired visual discrimination when they reached a performance criterion of at least 80% of trials correct (not including correction trials) on two consecutive 30-trial sessions. Mice were moved on to reversal learning individually, after they attained visual discrimination criterion. Reversal was identical to visual discrimination, except that S+ and S− were reversed. Criterion for completion of reversal learning was considered to be at least 80% correct on 2 consecutive sessions, but all mice received a minimum of 20 sessions regardless. Mice that did not attain criterion were tested for 25 sessions. The first two sessions of visual discrimination and reversal learning were each run over two days, in four sessions of 15 trials. These sessions were not counted towards the acquisition or reversal criteria.

Mice trained on visual discrimination and reversal learning were transferred to object-location paired-associates learning but were first subjected to the final phase of pretraining using a 3-hole mask. For paired-associates learning, three stimuli were used; flower, plane and spider. Whether a stimulus was correct was determined by the location in which it was presented (flower/left; plane/middle; spider/right). On each trial, one stimulus was presented in its correct location and one in its incorrect location resulting in 6 trial types^14^. Criterion for paired-associates learning acquisition was 70% correct on 2 consecutive sessions, but all mice received 50 sessions regardless.

Acquisition and extinction of a simple instrumental response^15^ was done after paired-associates learning, using the same 3-hole mask. Animals were required to touch a single white square in the central window and criterion was attained when 30 trials were finished in <14.5 min for 5 consecutive days. During the subsequent extinction phase, there was no requirement to initiate, but an initiation delay was implemented (5 s). The stimulus was presented in the central touchscreen window for 10 s. If the stimulus was not touched during this time (“omission”), it disappeared at the end of the 10 s, followed by a 5 s inter trial interval. If the stimulus was touched, it disappeared immediately and the 5 s inter trial interval began. Criterion for this phase of the task was ≥77% (23/30) omissions on 2 consecutive days, but mice were tested for a minimum of 10 sessions.

Finally, location discrimination was used to assess memory for screen locations and spatial pattern separation ability^13^,^16^ in a separate cohort of mice. Animals had to discriminate and choose between two illuminated (100% white) squares that were presented at small, intermediate or large distance (separation), within a row of 6 potential locations. Inactive locations were identifiable from the background of the screen through low illumination (10% white) and no mask was used. Mice were required to respond correctly in 7 out of 8 consecutive trials (acquisition) after which reward contingencies were reversed (reversal). Mice were first trained on intermediate separation (locations 2 and 5) until they could attain the overall acquisition criterion (perform one acquisition and one reversal within a session of maximum 60 trials, for 3 out of 4 consecutive days). Subsequently, pattern separation ability was assessed during probe sessions of unlimited trials, using only small separation (locations 3 and 4) or large separation (locations 1 and 6). In total, 12 probe sessions were applied, 6 of each separation in blocks of 2 sessions. Subsequently, we assessed whether mutant mice differ from wild-type mice at recall performance in the location discrimination (LD) task. For this, animals were re-trained on the intermediate separation in the absence of a within-session reversal during two consecutive days, in order to train animals to respond exclusively to one (i.e. either left or right) location on the screen. After this, mice were subjected to a 72-hour delay and retested on their ability to recall the previously rewarded location during a 40-minute recall session. Performance in this session was assessed in 10-trial bins.

### Adult neurogenesis and BrdU injections

In order to investigate differences in adult neurogenesis in the SGZ of the DG, proliferation rate of neural stem cells in *Ehmt1*^+/−^ mice (n = 7) was compared to that of WT animals (n = 8) and was assessed using BrdU and Ki-67 immunohistochemistry. For this, mice were intraperitoneally injected with 50 μg/g BrdU (Sigma, B9285) at postnatal day 70 ± 10 days for four times every 4 h during 12 h period, as previously described^24^. Mice were perfused transcardially, 24 h after the first BrdU injection with PFA 4% and sucrose 4% in PBS 0.1 M. Brains were removed and kept in the same perfusion solution o/n at 4 °C and then replaced with 0.1 M PBS with 0.025% NaN_3_. Coronal 30 μm thick sections throughout the hippocampus were cut with a Leica VT 1000 S vibratome. One in ten brain sections were collected for BrdU and Ki-67 immunohistochemistry and kept at 4 °C in 0.1 M PBS with 0.025% NaN_3_.

### BrdU and Ki-67 immunohistochemistry and stereological counting

For BrdU immunolabeling sections were first post fixed for 2 hrs in PFA 4%/sucrose 4% and washed in PBS 0.1 M/0.1% triton. Subsequently, the sections were immersed in pre-cooled 1 M HCl for 10 min at 4 °C, after 10 min in 2 M HCl at room temperature, and transferred for additional 10 min at 37 °C in the same solution. After this, sections were rinsed in 0.1 M boric acid (pH 8.5) for 12 min at room temperature. Subsequently, sections were washed and permeabilized in PBS 0.1 M with Triton 0.1% and then blocked with 10% Normal Goat Serum (NGS), 10% Normal Donkey Serum (NDS) in Phosphate Buffer Solution (PBS) 0.1 M/0.1% triton pH: 7.6 for 1 h at room temperature. Primary antibodies against BrdU (1:200, Abcam ab1893), Ki67 (1:50, BD Pharmigen 550609) and H3K9me2 (1:100, Millipore 07–449) in PBS 0.1 M/0.1% triton with 5% NGS and 5% NDS were incubated overnight at 4 °C. Brain sections were washed and incubated in Donkey anti-sheep Alexa Fluor 488 (1:1000, Invitrogen A11015) and Goat anti-Mouse 568 (1:1000, Invitrogen A11031) secondary antibodies in 5% NGS, 5% NDS in PBS 0.1 M/0.1% triton for 1 h at room temperature in darkness. Nuclear staining with Hoechst 33342 (1:5000, Invitrogen H3570) was performed after additional washing of secondary antibody excess in PBS 0.1 M/0.1% triton. Free-floating sections were then washed with PBS 0.1 M and mounted on a glass slide. Imaging was performed either with a Fluoview FV1000 confocal microscope (Olympus) for representative pictures or with a ZEISS IMAGER 2.1 epifluorescence microscope that was used for counting BrdU^+^ and Ki67^+^ cells. The total number of cells was calculated by counting the amount of either of BrdU^+^ or Ki67^+^ cells in the SGZ on eight coronal 30 μm brain sections. Every section was a cranio-caudal transition of 300 μm encompassing the entire length of the hippocampus, thus the total number of cells was calculated by the summing of the number of cells per section times 10^44^. Notably, there was no difference in size between WT and *Ehmt1*^+/−^ mice.

### EHMT1 immunostaining and brain preparation

A separate group of WT, n = 5 mice at postnatal 70 ± 10 days, were sacrificed by cervical dislocation and brains were snap frozen in liquid nitrogen and stored at −80 °C. Brains were embedded in the Shandon M-1 embedding matrix (Thermo Scientific). The brains were sectioned on a cryostat at −20 °C in the sagittal plane from medial to lateral and mounted on Superfrost Plus glass slides (Thermo Scientific). Brain sections were first fixated onto glass slides with ice-cold methanol for 10 min at −20 °C. After washing with 0.1 M Tris buffered saline pH: 7.6 with 0.05% Tween (TBS-T), slides were treated with 0.3% hydrogen peroxide for 10 min RT and washed in TBS-T. Ultra-Tek Superblock for 5 min was used to block nonspecific immunolabeling. Incubation with an EHMT1 primary antibody (1:100 Abcam, ab 41969) was conducted, initially for 30 min at room temperature and then continued overnight at 4 °C. The next day, the incubation box was left at room temperature for 30 min before washing with TBS-T. Thereafter, the slides were incubated with the Ultra-Tek anti-polyvalent staining system. After another 5 × 10 min washing steps with TBS-T, slides were incubated with Ultra/Tek HRP for 10 min. After washing with Tris buffer pH: 7.6 (TB), 0.05% 3,3′ diaminobenzidine tetrahydrochloride (DAB, Sigma) was added. After 10 min the DAB reaction was stopped by washing in TB, followed by washing in TBS. Dehydration was done in a series of alcohol dilutions (50%, 70%, 96%, 96%, 100%, 100%; each for 1 min). Then slides were treated with xylene and coverslipped with Pertex (Histolab Products AB, Göteborg, Sweden).

Analysis of EHMT1 immunostaining was performed using a bright light microscope (Leitz Dialux 22, Leitz Microscopes) and pictures were taken with a digital microscope camera (Nikon Coolpix 990, Nikon Corporation, Tokyo, Japan). EHMT1 intensity levels were compared between the SGZ and the granular cell layer in the DG in WT animals only (n = 5). We selected the SGZ by delineating a distance of one cell layer at the border of the hilus and granular cell layer. Mean density was measured by analyzing the intensity levels of EHMT1 staining in cell nuclei. Measurements were performed in 30 cells within the DG, 30 cells in the SGZ, and in the outer area for background density values. The values measured ranged between 0 (dark) and 256 (light), here reported as arbitrary units (a.u.). All the values were then transposed (i.e. subtracted from 256), in order to have a higher value corresponding to higher density. In this way, a dark stained cell was represented by a high-density value. These density values were then used to calculate the mean and standard error mean (SEM), and shown in tables and bar-graphs.

### Analysis & Statistics

#### Touchscreen operant training

For activity assessment during habituation, total beam breaks, chamber traversals and screen touches in 30 min were compared between genotypes. For pretraining, visual discrimination & reversal learning, paired-sssociates learning, extinction learning, the number of trials animals required to reach performance criterion was calculated for each mouse and averaged across genotypes. For extinction, it was necessary to exclude one mouse from the analysis after acquisition, due to the repeated escapes from the chamber, which resulted in WT n = 13, *Ehmt1*^+/−^ n = 10 (for acquisition) and n = 9 (for extinction). In addition to the ‘trials to criterion’ measure, for the Reversal learning paradigm the number of errors (incorrect choices on first presentation trials) and correction trials committed were analyzed, specifically during the early reversal phase (i.e. on sessions where animals performed below 50% correct). This was done to calculate an average “perseveration index” (PI) per animal; the ratio of CTs to incorrect responses on first presentation trials^45^. For location discrimination, the number of trials and errors mice required to reach the overall acquisition criterion of Location Discrimination training at intermediate distance were calculated. Furthermore, the number of trials mice required to attain within-session acquisition, but not reversal, are reported for small and large separation probes. This was done because the within-session reversal criterion was not reached consistently. In cases where animals had not reached the acquisition criterion, the total amount of trials performed within that session + 8 was used, as this would have been the minimum amount of trials needed to be able to reach that criterion. Based on anatomical MRI scans of mice within the cohort of animals that were tested in the location discrimination task, performed after the last day of behavioural testing (data not shown here), two *Ehmt1*^+/−^ mice were excluded from location discrimination analysis, as these animals showed, bilaterally, greatly enlarged ventricles and reduced hippocampi. In addition, two WT mice did not reach the overall criterion during initial acquisition at intermediate distance (after 40 sessions) and were excluded from further analysis, resulting in WT n = 17, *Ehmt1*^+/−^ n = 8. Finally, for both cohorts, the average reward collection latency and response latency were calculated.

#### Proliferation assay

For the analysis of cell proliferation, we counted the total number of either Ki67^+^ or BrdU^+^ cells in the DG per mouse. The number of cells positive for either marker was then averaged within the genotype to subsequently perform between genotypes statistical comparisons. For EHMT1 intensity analysis density values were then used to calculate the mean and standard error mean (SEM), and shown in tables and bar-graphs.

#### All experiments

Data are presented as the mean ± SEM. Where possible, data were analyzed using Student’s *t*-test for independent samples, one-sample *t*-test and repeated-measures ANOVA (within-subject factor: session block, between-subject factor: genotype), as appropriate. Where the assumption of homogeneity of variance was rejected (Levene’s test), an unequal variance *t*-test was used. Where the assumption of normality was rejected (Shapiro-Wilk test), attempts were made to normalize the distributions with appropriate transformations, but where data could not be normalized with transformations, the non-parametric Mann-Whitney U tests was used instead of the standard *t*-test. All ANOVA data were subjected to Mauchly’s test of sphericity to ensure that the homogeneity of variance assumption was not violated. Where it was, the Huynh-Feldt correction was used. All statistical analyses were conducted with a significance level of α = 0.05, using SPSS version 17.0.

## Additional Information

**How to cite this article**: Benevento, M. *et al*. Haploinsufficiency of EHMT1 improves pattern separation and increases hippocampal cell proliferation. *Sci. Rep.* **7**, 40284; doi: 10.1038/srep40284 (2017).

**Publisher's note:** Springer Nature remains neutral with regard to jurisdictional claims in published maps and institutional affiliations.

## Supplementary Material

Supplementary Information

## Figures and Tables

**Table 1:** Outcome-parameters of cognitive testing of *Ehmt1* ^+/−^ mice in learning and memory tasks in the touchscreen operant chamber.

Task	Outcome behavioural phenotype	Performance (*)	Significance
Pretraining Visual Discrimination	Normal	WT 8.8 ± 0.5 *Ehmt1*^+/−^ 10.7 ± 0.8	p > 0.1
Pretraining Location Discrimination	Normal	WT 25.0 ± 1.0 *Ehmt1*^+/−^ 23.0 ± 1.1	p > 0.1
Visual Discrimination	Normal	WT 249.2 ± 37.2 *Ehmt1*^+/−^ 234.0 ± 29.6	p > 0.1
Reversal Learning	Normal	WT 549.2 ± 55.6 *Ehmt1*^+/−^ 492.0 ± 63.0	p > 0.1
Reversal Learning early phase	Higher perseverative index (PI) in *Ehmt1*^+/−^ mice	WT 3.5 ± 0.2 (PI) *Ehmt1*^+/−^ 4.9 ± 0.5 (PI)	p = 0.009
Object-location Paired-Associates Learning	Normal	WT 1182 ± 127 *Ehmt1*^+/−^ 1422 ± 128	p > 0.1
Acquisition Instrumental Response	Normal	WT 163.8 ± 7.3 *Ehmt1*^+/−^ 177.0 ± 9.4	p > 0.1
Extinction Instrumental Response	Normal	WT 200.8 ± 18.4 *Ehmt1*^+/−^ 206.7 ± 31.4	p > 0.1

(*) Performance is reported as the average number of trials required to reach the task-criterion for all tests, with the exception of Reversal learning - early phase, in which the perseverative index is reported (PI). PI = total number of errors made/number of first presentation errors.
