# Supplementary material for: Haploinsufficiency of EHMT1 improves pattern separation and increases hippocampal cell proliferation
Source: Sci Rep. 2017 Jan 10;7:40284. doi: 10.1038/srep40284 (PMC5223204; doi:10.1038/srep40284)
Supplement: Supplementary Information [file srep40284-s1.pdf]

**Haploinsufficiency of EHMT1 improves pattern separation and increases hippocampal cell proliferation.**

Marco Benevento[1*], Charlotte A. Oomen[1*], Alexa E. Horner[2,3,4,&], Houshang Amiri[1,5], Tessa Jacobs[1], Charlotte Pauwels[1], Monica Frega[1], Tjitske Kleefstra[8], Maksym V. Kopanitsa[2,¶], Seth G. N. Grant[6], Timothy J. Bussey[3,4], Lisa M. Saksida[3,4], Catharina E.E.M. Van der Zee[7], Hans van Bokhoven[1,8], Jeffrey C. Glennon[1], Nael Nadif Kasri[1,8,↑]

[1]Department of Cognitive Neuroscience, Radboudumc, Donders Institute for Brain, Cognition and Behaviour, Nijmegen, the Netherlands
[2]Synome Ltd., Babraham Research Campus, Cambridge CB22 3AT, UK
[3]Department of Psychology, University of Cambridge, Cambridge, UK
[4]Behavioural and Clinical Neuroscience Institute, University of Cambridge, Cambridge, UK;
[5]Neuroscience Research Center, Institute of Neuropharmacology, Kerman University of Medical Sciences, Kerman, Iran
[6]Centre for Clinical Brain Sciences, Chancellors Building, University of Edinburgh, 49 Little France Crescent, Edinburgh EH16 4SB, UK
[7]Department of Cell Biology, Radboudumc, Radboud Institute for Molecular Life Sciences, Nijmegen, the Netherlands
[8]Department of Human Genetics, Radboudumc, Donders Institute for Brain, Cognition, and Behaviour, Nijmegen, the Netherlands

[&]Present address: Cambridge Admissions Office, University of Cambridge, Fitzwilliam House, 32 Trumpington Street, Cambridge CB2 1QY, UK
[¶]Present address: Charles River Discovery Research Services, Microkatu 1, 70210 Kuopio, Finland
[*]Equal contribution.

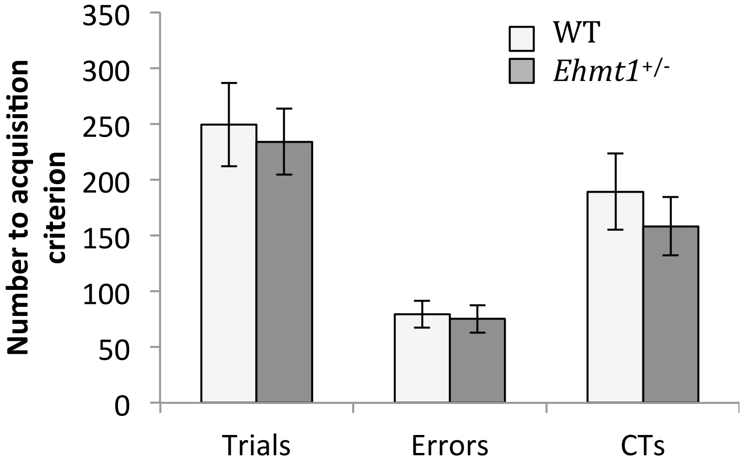


**Figure S1. Visual discrimination (VD) performance**

Performance of *Ehmt1*$^{+/-}$ and WT mice on VD acquisition, in terms of the number of trials, errors and correction trials (CTs) to criterion. Data presented as mean ± SEM. WT n =13, *Ehmt1*$^{+/-}$ n = 10. No significant differences between the groups were observed. Note that for VD no day-to-day acquisition averages exist, as the number of days mice required to reach criterion differed and animals were moved to Reversal learning individually

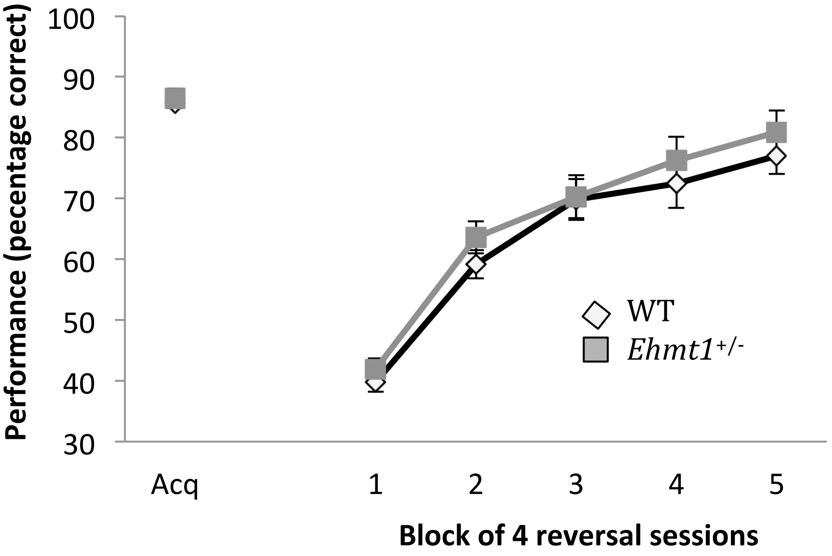


**Figure S2. Reversal learning - acquisition graph.**

Performance of *Ehmt1*$^{+/-}$ and WT mice on VD Reversal, in terms of performance (percentage correct) during the first 20 sessions of reversal, in blocks of 4 sessions. Acquisition ("Acq") data point is the average of each mouse's last two acquisition sessions. Data presented as mean ± SEM. WT n =13, *Ehmt1*$^{+/-}$ n = 10. Analysis of reversal across blocks of 4 sessions revealed a significant effect of block ($p < 0.001$), no effect of interaction or genotype ($p > 0.1$).

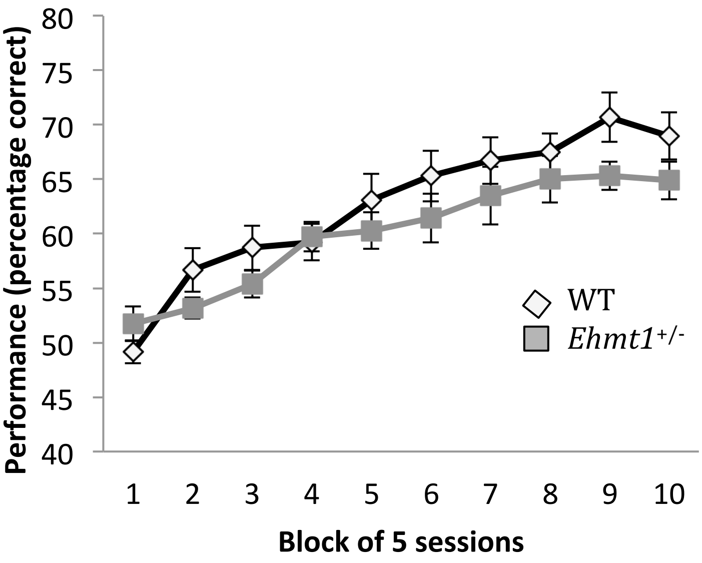


**Figure S3. Object-place paired associates learning (PAL) acquisition graph.** Performance (percentage correct) of WT (n = 13) and *Ehmt1*$^{+/-}$ (n = 10) mice during PAL acquisition, in blocks of 5 sessions. Data presented as mean ± SEM. Analysis of PAL acquisition revealed a significant main effect of block ($F_{9,189} = 33.957$, $p < 0.001$; Figure X), but no effect of genotype ($F_{1,21} = 1.575$, $p > 0.1$) or interaction of these factors ($F_{9,189} = 1.398$, $p > 0.1$).

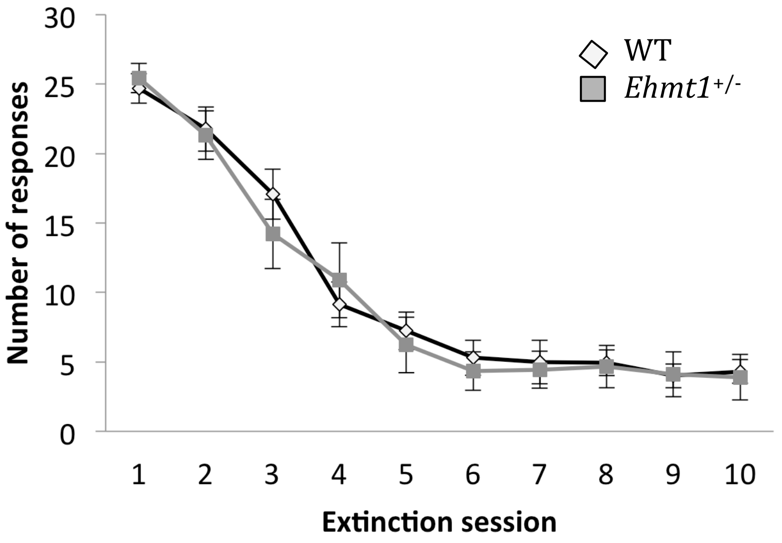


**Figure S4. Extinction learning acquisition graph.**

Performance (number of responses out of a possible 30) during the first 10 sessions of an appetitive instrumental extinction task. Data presented as mean ± SEM. WT n = 13; *Ehmt1*$^{+/-}$ n = 10. Responding during the first 10 sessions of extinction revealed a significant main effect of session (Huynh-Feldt-corrected RM ANOVA; $F_{6.394,\ 127.883}$ = 73.415, $p < 0.001$), indicating that responding reduced with training. However, there was no significant effect of genotype or of an interaction between these factors (both $F < 1$, $p > 0.1$).

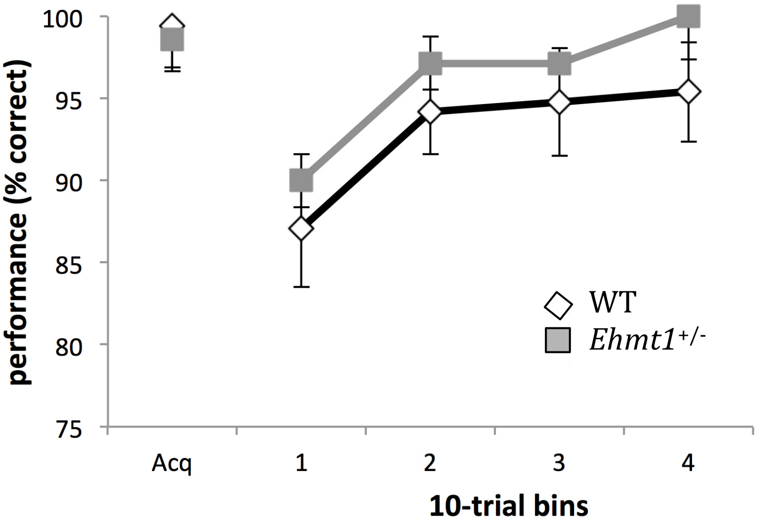


**Figure 5 Location Discrimination recall test**

Performance (percentage correct) on the recall session in the Location Discrimination test. Mice first acquired the location-reward contingency during three sessions without reversal (Acq; average of the final acquisition session) and were then re-tested after a 72 hour delay.Data depicted in bins of 10 trials and presented as mean ± SEM. Both groups show a mild drop in performance during the first 10-trial bin, however, no significant effect of genotype or of an interaction between genotype and bin was found (both $F < 1$, $p > 0.1$).
